# Design and Preclinical Evaluation of a Nanoparticle Vaccine against Respiratory Syncytial Virus Based on the Attachment Protein G

**DOI:** 10.3390/vaccines12030294

**Published:** 2024-03-12

**Authors:** Richard Voorzaat, Freek Cox, Daan van Overveld, Lam Le, Lisanne Tettero, Joost Vaneman, Mark J. G. Bakkers, Johannes P. M. Langedijk

**Affiliations:** Janssen Vaccines & Prevention BV, 2333 CN Leiden, The Netherlands

**Keywords:** RSV, lumazine synthase, attachment protein G, nanoparticle, vaccine

## Abstract

Human respiratory syncytial virus (RSV) poses a significant human health threat, particularly to infants and the elderly. While efficacious vaccines based on the F protein have recently received market authorization, uncertainties remain regarding the future need for vaccine updates to counteract potential viral drift. The attachment protein G has long been ignored as a vaccine target due to perceived non-essentiality and ineffective neutralization on immortalized cells. Here, we show strong G-based neutralization in fully differentiated human airway epithelial cell (hAEC) cultures that is comparable to F-based neutralization. Next, we designed an RSV vaccine component based on the central conserved domain (CCD) of G fused to self-assembling lumazine synthase (LS) nanoparticles from the thermophile *Aquifex aeolicus* as a multivalent antigen presentation scaffold. These nanoparticles, characterized by high particle expression and assembly through the introduction of N-linked glycans, showed exceptional thermal and storage stability and elicited potent RSV neutralizing antibodies in a mouse model. In conclusion, our results emphasize the pivotal role of RSV G in the viral lifecycle and culminate in a promising next-generation RSV vaccine candidate characterized by excellent manufacturability and immunogenic properties. This candidate could function independently or synergistically with current F-based vaccines.

## 1. Introduction

Human respiratory syncytial virus (RSV) is an enveloped, negative-sense single-stranded RNA virus belonging to the *Pneumoviridae* family. It is among the leading causes of lower respiratory tract infections, including severe bronchiolitis and pneumonia, in infants as well as the elderly and immune-compromised individuals [1,2]. 

**Figure 1:**
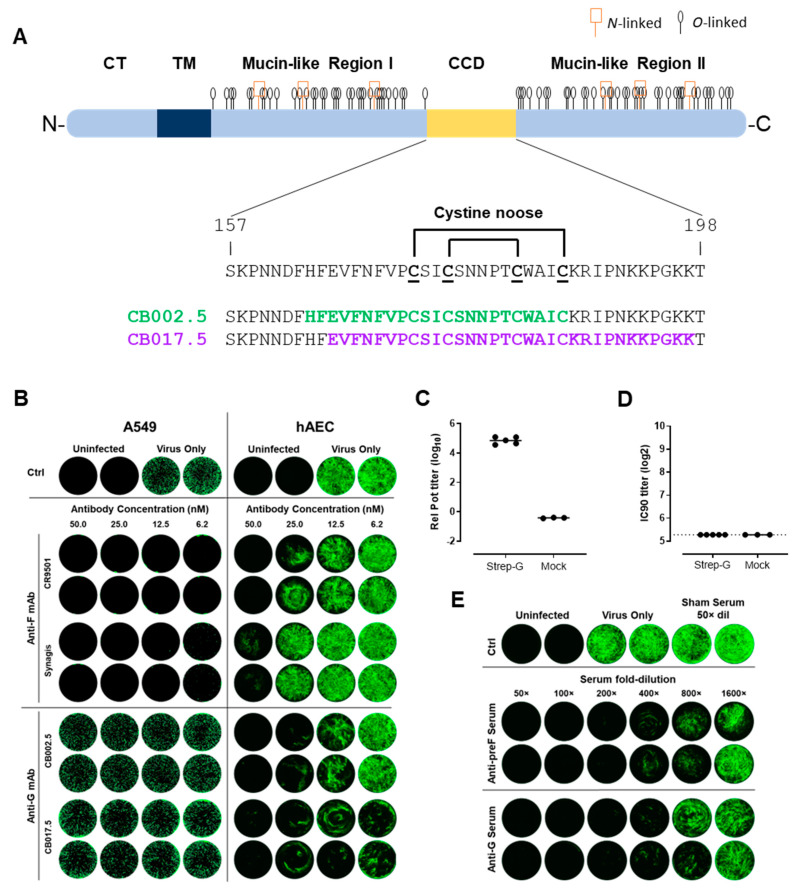
Antigenic overview of RSV G. (**A**) Schematic of hRSV G with labeled domains. N- and O-linked glycosylation sites are indicated on the ectodomain. Zoom-in between amino acid positions 157 and 198 of the cystine noose is indicated. The epitopes of anti-RSV G monoclonal antibodies CB002.5 and CB017.5 are shown on the CCD amino acid sequence in green and magenta, respectively. CT: cytoplasmic tail, TM: transmembrane, CCD: central conserved domain. (**B**) RSV A2-GFP reporter gene VNA using anti-RSV F or G monoclonal antibody on A549 cells (**left panel**) and hAEC cultures (**right panel**). Infected cells turn green by reporter protein expression. (**C**–**E**) Preclinical evaluation of a proof-of-concept RSV G particle-based vaccine. (**C**) CCD binding antibody titers determined by ELISA using immobilized CCD peptides and displayed as log10 relative potency (rel pot). (**D**) RSV A neutralizing antibody titers determined by VNA using firefly luciferase (FFL) labeled RSV strain and A549 cells and displayed as log2 of the 90% FFL inhibitory serum dilution (IC90) in serum collected on day 42 from mice (*n* = 3 or 5) immunized on day 0 and 21 with buffer or with biotinylated G peptide coupled to streptavidin (Strep-G) and adjuvanted with 2% Adjuplex. Bars represent mean responses per group. (**E**) VNA titers using RSV A expressing a GFP reporter on hAEC cultures with pooled serum samples of Strep-G (**C**) or preF protein-immunized mice.

RSV attachment and entry is governed by two virus-encoded surface glycoproteins: the fusion protein F and the attachment protein G. F is a trimeric class I fusion glycoprotein that mediates fusion of viral and host cell membranes. RSV G is a tetrameric type 2 transmembrane protein, consisting of a central conserved domain (CCD) flanked by two mucin-like regions [3]. The CCD is non-glycosylated and comprises a cystine noose with two disulfide bridges and a heparin binding domain (Figure 1A) [3,4,5]. The G protein is thought to mediate the initial attachment to the host cell by binding to the CX3C receptor CX3CR1 [4,5,6,7,8,9,10]. G-mediated attachment is not critical for in vitro infection, as ΔG RSV is able to infect cells [11]. However, infection efficiency is increased 600-fold when G is present during infection of fully differentiated human airway epithelial cell (hAEC) cultures; additionally, no RSV strains lacking G have been isolated from patients [12]. 

Viral surface proteins make for attractive vaccine targets as they often play pivotal roles during viral entry and are exposed to the humoral immune system. Protective RSV vaccines have recently been approved based on the fusion protein F stabilized in its prefusion conformation (preF) [13,14]. Currently, it is not clear how many seasons their protective efficacy will last and whether immune pressure will cause the F protein to evolve and undergo antigenic drift. The realization that the efficacy of viral vaccines does not necessarily depend on a strong response against the fusion protein was recognized for both influenza, where vaccine efficacy declined when the neuraminidase content was low [15], and for the paramyxovirus Hendra virus, for which an efficacious vaccine is approved based solely on the attachment protein H [16,17]. Although the G protein has been undervalued compared to F, the recent demonstration of strong neutralization in hAEC cultures of antibodies against G show the potential as a vaccine component [5,18,19,20], and protection from RSV challenge was demonstrated in mice after immunization with G [21,22,23,24,25]. Therefore, if durability and breadth of immunity elicited by current F-based RSV vaccines may turn out to be insufficient, inclusion of an additional RSV vaccine component based on the attachment protein G might increase the chance of durable and broad protection.

Induction of an effective human immune response benefits from multivalent, repetitive display of the antigen since this allows for more effective cross-linking of B cell receptors and B cell activation and proliferation in comparison to monomeric immunogens [26,27,28]. Lumazine synthase (LS) is a bacterial enzyme that is part of the riboflavin biosynthesis pathway [29]. LS from the thermophile *Aquifex aeolicus* (AaLS) forms pentameric protein complexes that assemble into icosahedral capsids by non-covalent interactions between 12 pentamers, ultimately forming a hollow nanoparticle (NP) with a diameter of around 16 nm composed of 60 subunits [29,30]. With the N- and C-termini of the monomers outward facing, peptide antigens can be genetically fused to these termini to display them to the immune system [29,31]. Given their thermophile origins, AaLS NPs are conformationally stable and resilient to high temperatures. These characteristics, combined with the excellent safety profile in animals and humans, make AaLS an attractive scaffold for antigen presentation [32].

Here, we show that the strong neutralizing potential of RSV G-targeting antibodies is on par with F-targeting antibodies, but these antibodies could only be evaluated in primary, fully differentiated hAEC cultures and not immortalized cell lines. To investigate G as a vaccine antigen, we designed and characterized one-component self-assembling nanoparticles displaying the RSV G CCD peptides by introducing N-linked glycosylation sites on the surface of AaLS to achieve particle expression and assembly. Preclinical evaluation in a mouse model showed induction of neutralizing G-targeting antibodies as determined in neutralization assays with hAEC cultures. The AaLS nanoparticle presents a next-generation RSV vaccine candidate with excellent antigenic and immunogenic characteristics that is ready for further clinical evaluation.

## 2. Materials and Methods

### 2.1. AaLS-RSV G-CCD Design and Screening

Self-assembling *Aquifex aeolicus* lumazine synthase (AaLS) nanoparticle variants equipped with a human CD5 signal peptide were cloned into a pcDNA2004 plasmid DNA and produced by Genscript (Piscataway, NJ, USA). The initiating methionine of the AaLS gene was removed to prevent translation initiation after the signal peptide sequence, thus preventing cytosolic retention of the protein. hRSV and bRSV G CCD sequences (Table S1) were genetically fused to the N-terminus, C-terminus, or the internal 70/71 loop of the AaLS particle. Varying repeats of N-linked glycosylation sites were added to the permissive internal loop of lumazine synthase to aid expression and solubility. Designs were screened in the Expi293 expression system (Gibco, Thermo Fisher Scientific, Bleiswijk, The Netherlands). Expi293F cells were transiently transfected at a 200 μL scale at a cell density of 2.5 × 10^6^ vc/mL using the ExpiFectamine 293 Transfection Kit (Gibco, Thermo Fisher Scientific, Bleiswijk, The Netherlands) according to the manufacturer’s instructions in Expi293F Expression medium [+] GlutaMAX (Gibco, Thermo Fisher Scientific, Bleiswijk, The Netherlands) supplemented with 50 U/mL Pen-Strep (Gibco, Thermo Fisher Scientific, Bleiswijk, The Netherlands) in 96-half deep-well plates. Following three days of culture at 37 °C, 75% humidity, and 8.0% CO_2_ and while under constant agitation at 250 RPM, the cell supernatant was harvested with centrifugation for 10 min at 500× *g* and subsequent 96-well spin-filter clarification using AcroPrep Advance Supor Filter plates (0.2 µm, Pall, Cytiva, Uppsala, Sweden) for 10 min at 500× *g*. Clarified supernatant was stored at 4 °C until further use. Expression levels and the oligomeric state were assessed using analytical size-exclusion chromatography SEC.

### 2.2. Production and Purification of AaLS Nanoparticles

Expi293F cells (Gibco, Thermo Fisher Scientific, Bleiswijk, The Netherlands) were transiently transfected at a 300 mL scale at a cell density of 2.5 × 10^6^ vc/mL using the ExpiFectamine 293 Transfection Kit (Gibco, Thermo Fisher Scientific, Bleiswijk, The Netherlands) according to the manufacturer’s instructions in Expi293F Expression medium [+] GlutaMAX (Gibco, Thermo Fisher Scientific, Bleiswijk, The Netherlands) in 1 L vented flasks. One day after transfection, enhancers were added. Following five days of culture at 37 °C, 75% humidity, and 8.0% CO_2_ and while under constant agitation at 125 RPM, the cell supernatant was harvested using centrifugation for 10 min at 600× *g* and subsequently filtered using a 0.22 µm PES vacuum filter (Nalgene, Thermo Fisher Scientific, Bleiswijk, The Netherlands) to obtain clarified sterile supernatant. The clarified supernatant was stored at 4 °C until further use. 

C-tagged AaLS particles trimers were purified using a three-step protocol on an ÄKTA Avant 25 system (Cytiva, Uppsala, Sweden). Briefly, 0.22 µm filtered supernatant was applied to a 5 mL capture Select C-tag XL column (Thermo Fisher Scientific, Bleiswijk, The Netherlands) equilibrated in PBS pH 7.4 (1×, Gibco, Thermo Fisher Scientific, Bleis-wijk, The Netherlands). Then, the column was washed with PBS, and the protein was eluted with 20 mM Tris, 2 M MgCl2, pH 7.0 and 1:1 diluted with 20 mM Tris, pH 8.0 to lower the MgCl2 concentration. The obtained protein was concentrated using an Amicon Ultra 15 50-kDa cutoff filter (Millipore, Merck Life Science, Amsterdam, The Netherlands) prior to further purification via size exclusion chromatography. The protein was applied on a HiLoad Superdex 200 16/600 column (Cytiva, Uppsala, Sweden) equilibrated in 20 mM Tris, 75 mM NaCl, pH 7.4, and all fractions containing high molecular weight protein were pooled and concentrated using an Amicon Ultra 15 50-kDa cutoff filter. Subsequently, the protein was applied to a Superose 6 10/300 GL column (Cytiva, Uppsala, Sweden) equilibrated in 20 mM Tris, 75 mM NaCl, pH 7.4, and monodisperse fractions were pooled. Sucrose was added to a final concentration of 5%, and the pool was filtered (0.22 µm) to form the final product. Nanoparticles were either stored at 4 °C or snap frozen in liquid nitrogen for long term storage at −80 °C.

### 2.3. Analytical SEC and SEC-MALS on AaLS Nanoparticles

Analytical SEC on clarified supernatant was performed by applying 20 μL sample to a SRT-C C-500A 15 cm column (Sepax Technologies, Newark, DE, USA) with the corresponding guard column (Sepax Technologies, Newark, DE, USA) at 25 °C equilibrated in running buffer (150 mM sodium phosphate, 50 mM NaCl, pH 7.0) at 0.35 mL/min on an ultra-high-performance liquid chromatography system (Vanquish, Thermo Fisher Scientific, Bleiswijk, The Netherlands). Analytical SEC data were analyzed using Chromeleon 7.2.8.0. OD280 UV traces of mock transfections were used to perform baseline correction. 

Purified protein was analyzed by using an ultra-high-performance liquid chromatography system (Vanquish, Thermo Fisher Scientific, Bleiswijk, The Netherlands) and µDAWN TREOS instrument (Wyatt, Santa Barbara, CA, USA) combined with an Optilab µT-rEX Refractive Index Detector (Wyatt, Santa Barbara, CA, USA) and coupled with an in-line Nanostar DLS reader (Wyatt, Santa Barbara, CA, USA). A maximum of 10 μg protein or 20 μL sample volume was analyzed by applying the sample to a SRT-C C-500A 15 cm column (Sepax Technologies) with the corresponding guard column (Sepax Technologies, Newark, DE, USA) at 25 °C equilibrated in running buffer (150 mM sodium phosphate, 50 mM NaCl, pH 7.0) at 0.35 mL/min on an ultra-high-performance liquid chromatography system (Vanquish, Thermo Fisher Scientific, Bleiswijk, The Netherlands). The obtained data were analyzed using the Chromeleon 7.2.8.0 software package, and the hydrodynamic radius, conformation, and molecular weight of AaLS particles were calculated with Astra 8.0.0.19 software (Wyatt, Santa Barbara, CA, USA) using a dn/dc value of 0.185 for the protein component and 0.1410 for the glycan component, per the manufacturers’ recommendation. Molecular weights were calculated using the RI detector as the source for concentration and mass recoveries and UV as the source for concentration.

### 2.4. NS-TEM

Negative stain transmission electron microscopy (NS-TEM) was performed at Ne-CEN (Leiden, The Netherlands). Purified protein samples were diluted in 20 mM Tris, 75 mM NaCl, pH 7.4 and stained for 30 s with 2% uranyl acetate. The final protein concentration was 20 µg/mL. Following the staining procedure, the samples were applied to carbon film 200 mesh copper grids (Quantifoil, Großlöbichau, Germany) that underwent glow discharging at 25 mA for 45 s in an Pelco easiGLow (Ted Pella, Inc., Redding, CA, USA) prior to use. Micrographs were collected on a Talos L120C operated at 120 kV (Thermo Fisher Scientific, Bleiswijk, The Netherlands) at a resolution of 2.4 Ångstrom per pixel.

### 2.5. DLS

Purified protein was diluted in 20 mM Tris, 75 mM NaCl, pH 7.4 to a final concentration of 0.5 mg/mL. Of the diluted sample, 9 μL was loaded in a Uni-well (UNchained Labs, Malvern, UK) and analyzed with the sizing and polydispersity function (dynamic light scattering) in an UNcle (UNchained Labs, Malvern, UK). Each measurement consisted of 10 runs, each lasting 5 s, at 30 °C to obtain the Z-average diameter and polydispersity index (PDI) as a read-out. 

### 2.6. DSF

PBS pH 7.4 (1×, Gibco, Thermo Fisher Scientific, Bleiswijk, The Netherlands) was added to 20 μg of purified protein to a total volume of 90 uL to which 10 µL 50x SYPRO Orange (5000× stock diluted in PBS, Thermo Fisher Scientific, Bleiswijk, The Netherlands) was added. A negative control sample containing no protein was included for reference subtraction and stabilized RSV preF with a known Tm50 was used as positive control. Triplicates of 30 µL were dispensed in a MicroAmp Fast Optical 96-well Plate (Thermo Fisher Scientific, Bleiswijk, The Netherlands) and sealed with MicroAmp Optical Adhesive Film (Thermo Fisher Scientific, Bleiswijk, The Netherlands). The samples were heated using a temperature ramp from 25 to 95 °C with a rate of 0.015 °C per second. Datapoints were collected continuously with the qPCR instrument (Applied Biosystems ViiA 7, Thermo Fisher Scientific, Bleiswijk, The Netherlands) measuring reporter ROX. The melting temperatures were derived from the negative first derivative, which was plotted as a function of temperature. The lowest point in the curve indicated the melting temperature.

### 2.7. Freeze–Thaw

Purified proteins in 20 mM Tris, 75 mM NaCl, pH 7.4 were subjected to freeze–thaw (FT) cycles in the presence or absence of sucrose. To the samples with sucrose, sucrose from a 50% stock was added to a final concentration of 5% (*v*/*v*). Volumes were made in such a way that 20 µL could be retrieved for each measuring point. The total volume was snap-frozen in liquid nitrogen, after which it was thawed at room temperature. Samples were taken after 0× FT, 1× FT, and 5× FT from the same vial. Collected samples were analyzed using SEC-MALS as described above. Recovery rates were plotted against the amount of FT cycles.

### 2.8. Heat-SEC on Purified Proteins

Purified protein was diluted in 20 mM Tris, 75 mM NaCl, pH 7.4 to a final concentration of 0.5 mg/mL. Per temperature point, 45 μL of diluted protein was aliquoted in a 500 μL Eppendorf tube. The protein was heated at 60 °C, 70 °C, 80 °C, or 90 °C (ThermoMixer C, Eppendorf, Nijmegen, The Netherlands) for 30 min, 0 rpm, whereas the control was kept at 4 °C. Subsequently, the samples were centrifuged at 18,000× *g* for 10 min to remove large aggregates. Read-out was performed using SEC-MALS as described above. 

### 2.9. Western Blot

Proteins were separated on size using SDS-PAGE on Bolt 4–12% (*w*/*v*) Bis-Tris plus mini protein gels (Thermo Fisher Scientific, Bleiswijk, The Netherlands) in 1× MOPS running buffer under reducing conditions and blotted to PVDF membranes using the iBlot2 system (Thermo Fisher Scientific, Bleiswijk, The Netherlands). Manufacturers’ instructions were followed during all procedures. Blots were probed using a biotin anti-C-tag single domain antibody fragment conjugate (Thermo Fisher Scientific, Bleiswijk, The Netherlands) followed by IRDye 800CW-conjugated streptavidin (LI-COR Biotechnology, Bad Homburg, Germany). Bands were visualized on an Odyssey M imaging system (LI-COR Biotechnology, Bad Homburg, Germany), and scans were analyzed using Empiria Studio 3.0 (LI-COR Biotechnology, Bad Homburg, Germany).

### 2.10. Ethical Statement

Mouse studies were carried out at Janssen Vaccines and Prevention B.V., in compliance with the Dutch Animal Experimentation Act and the Guidelines on the Protection of Animals for scientific purposes set forth by the Council of the European Committee. These studies received approval from the Centrale Commissie Dierproeven and the Dier Experimenten Commissie of Janssen Vaccines and Prevention B.V.

### 2.11. Immunogenicity of Strep-G and AaLS-G Particles in Mice

Female BALB/c mice (Charles River, Lyon, France), 6–8 weeks of age, were intramuscular immunized on day 0 and day 21 with 2% Adjuplex adjuvanted biotinylated G-CCD peptide coupled to streptavidin tetramers as a scaffold (strep-G), 2% Adjuplex adjuvanted 1 or 10 µg AaLS displaying RSV G CCD peptide (*n* = 5 mice), or formulation buffer only (*n* = 3 mice) in a total volume of 100 µL/mouse. Intermediate blood samples were taken at day −1 and 20 via the submandibular route. Animals were sacrificed on day 42, which was 2 weeks after the second immunization, and exsanguinated by heart puncture. 

N-terminally biotinylated RSV G-CCD-encoding peptides (Table S1) were ordered from PepScan (Lelystad, The Netherlands) and coupled in a 1:1 molar ratio to streptavidin from *Streptomyces avidinii* (Merck Life Science, Amsterdam, The Netherlands) in 20 mM Tris with 150 mM NaCl, pH 7.0 for 1 h at 37 °C under constant agitation.

### 2.12. G-CCD Binding Antibody Responses Measured by ELISA

G-CCD binding antibody titers were measured by ELISA in serum samples collected on days 21 and 42. Biotinylated RSV A G-CCD peptides (Table S1) were bound to 96-wells plates (Greiner Bio-one, Alphen aan den Rijn, The Netherlands) coated with avidin (for streptavidin immunizations) or streptavidin (for AaLS immunizations) (Merck Life Science, Amsterdam, The Netherlands). Unbound peptide was washed away using 1× PBS (Thermo Fisher Scientific, Bleiswijk, The Netherlands) with 0.05% Polysorbate-20 (Merck Life Science, Amsterdam, The Netherlands). The assay plates were blocked using 2% BSA (Merck Life Science, Amsterdam, The Netherlands) in 1× PBS with 0.05% Polysorbate-20 for 30 min at room temperature. Mouse serum was three-fold serial diluted in 1% BSA in 1× PBS with 0.05% Polysorbate-20 and allowed to bind to the coated peptides for 1 h at room temperature. Unbound antibodies were washed away using 1× PBS with 0.05% Polysorbate-20. Following the wash step, all plates were incubated with goat anti-Mouse IgG-HRP (BioRad, Lunteren, The Netherlands) for 1 h. Unbound secondary antibody was washed away using 1× PBS with 0.05% Polysorbate-20. Assay plates were developed using LumiGLO substrate (KPL, LGC SeraCare, Milford, MA, USA), and luminescent signals were measured using a Synergy Neo Plate Reader (Agilent Technologies, Santa Clara, CA, USA). Titers relative to MAB858-2 (Merck Life Science, Amsterdam, The Netherlands), added as a standard to every plate, were calculated and plotted as relative potency (Rel Pot) log10.

### 2.13. Virus Neutralization Assay (VNA) Using Monolayer Cell Culture

A549 or HeLa cells were seeded in 24-well tissue culture plates (Corning, Merck Life Science, Amsterdam, The Netherlands) in infection medium (1× DMEM; Gibco, Thermo Fisher Scientific, Bleiswijk, The Netherlands) supplemented with 50 U/mL Pen-Strep (Gibco, Thermo Fisher Scientific, Bleiswijk, The Netherlands) and 5% fetal bovine serum (Gibco, Thermo Fisher Scientific, Bleiswijk, The Netherlands) and cultured at 37 °C, 10% CO_2_ until 60 to 80% confluency was reached. Anti-RSV F or G monoclonal antibodies were serially diluted in infection medium and mixed 1:1 with RSV A2-GFP virus (ViraTree, Durham, NC, USA) diluted to 6.000 TCID50 units/mL in infection medium. The medium on the monolayer cells was replaced with 0.5 mL monoclonal antibody–virus mixture, and the plates were incubated for 4 days at 37 °C, 10% CO_2_. GFP fluorescent signals were visualized using a BioTek Cytation 1 automated microscope (Agilent Technologies, Santa Clara, CA, USA) using a 2.5× plan achromat objective (Meiji Techno, Campbell, CA, USA). The individual images were stitched using the Gen5i Plus v3.08.01 software package.

### 2.14. Automated VNA Using RSV A Strain CL57-FireFly 

Animal serum samples were heat inactivated and serially diluted. The diluted samples were then combined with 2.5 × 10^4^ pfu of firefly luciferase (FFL)-labeled RSV CL57, which was propagated in A549 cells obtained from the American Type Culture Collection (Manassas, VA, USA) following an incubation at room temperature (RT) for 1 h. Next, 5 × 10^3^ A549 cells were added to each well, and the plates were incubated at 37 °C in 10% CO_2_ for 20 h. After the 20 h incubation period, Neolite substrate from PerkinElmer (Shelton, CT, USA) was introduced, and the luminescence signal was measured using either an Envision plate reader from PerkinElmer (Shelton, CT, USA) or a BioTek Synergy NEO plate reader from Agilent Technologies (Santa Clara, CA, USA). The virus neutralizing titers were determined based on the 90% inhibitory concentration (IC90).

### 2.15. hAEC Culture VNA

Fully differentiated human airway epithelial cells (hAEC) of nasal origin (MucilAir) and grown on an air–liquid interface were purchased from Epithelix Sàrl (Geneva, Switzerland). The inserts consisted of cells from 14 anonymized donors. The neutralizing capacity of monoclonal antibodies and sera were tested in hAECs infected with RSV A2-GFP, which was purchased from ViraTree (Durham, NC, USA). Ready-to-use MucilAir inserts were maintained at an air–liquid interface according to the manufacturer’s instructions for 4 days prior to the start of the experiment. Each hAEC insert had undergone prior testing by the manufacturer to ensure ciliary beating, polarization of the epithelial layer, and mucus production, corresponding to healthy respiratory epithelium. At the start of the experiment, inserts were washed once with 200 μL 1× PBS (Thermo Fisher Scientific, Bleiswijk, The Netherlands) to remove mucus and cell debris. Cells were infected using 3.000 TCID50 units of RSV A2-GFP diluted to a final volume of 25 μL 1× PBS and gently mixed 1:1 with 25 μL antibody or serum dilution before being added to the apical side of the inserts. After a 1 h incubation at 37 °C, the antibody or serum/virus mixture was aspirated. Negative controls were infected in presence of a 50-fold dilution of serum from mock-vaccinated mice. Inserts were incubated for 96 h at 37 °C post-infection before the GFP fluorescent signal was visualized using a BioTek Cytation 1 automated microscope (Agilent Technologies, Santa Clara, CA, USA) with a 2.5× plan achromat objective (Meiji Techno, Campbell, CA, USA). The individual images were stitched using the Gen5i Plus v3.08.01 software package.

## 3. Results

### 3.1. RSV G CC Is a Potent Target for Neutralizing Antibodies

To evaluate the CCD of RSV G as a potential vaccine target, human monoclonal antibodies (mAbs) that target the CCD were tested in a virus neutralization assay (VNA) with A549 cells, an immortalized cell line of human lung epithelial origin, and primary, fully differentiated human airway epithelial cells (hAECs) (Figure 1A,B). Since RSV PreF is a proven efficacious vaccine target, human mAbs against F and G were tested in both types of VNA to evaluate the potential for RSV G as an additional vaccine target. MAbs included CB002.5 and CB0017.5 directed to the CCD of RSV G [5]; F-binding antibodies, Synagis (palivizumab), directed to site II and used prophylactically in newborns; and CR9501 directed to sites Ø/V [33,34]. In line with previous findings, anti-F mAbs effectively neutralized RSV in A549 and HeLa cells, but anti-G mAbs did not (Figure 1B and Figure S1). Strikingly, both G mAbs potently neutralized RSV when a VNA was performed using hAEC cultures and even outperformed the F-targeting mAbs. These results suggest that the potential of G-based neutralization is underestimated when immortalized cell lines are used that do not mimic the authentic virus attachment and entry route. In fact, RSV attachment to and entry into immortalized cells have been reported to occur by binding to cell surface heparan sulfate, negatively charged polysaccharide chains that are ubiquitously present on cell lines, but are reported to be absent in hAEC cultures [35,36,37]. Indeed, when we prevented heparan sulfate biosynthesis with CRISPR/Cas9-mediated knock-out of ß1,4-galactosyltrasferase 7 (B4GALT7), one of the key enzymes in the pathway, HeLa cells were rendered unsusceptible to RSV infection (Figure S2). Importantly, our results show that two, relatively randomly selected, CCD-specific mAbs show more potent neutralization compared with a highly potent neutralizing antibody against site Ø/V.

Next, to test whether G CCD could also induce RSV neutralizing antibodies in a preclinical setting, Balb/c mice were immunized with biotinylated CCD peptides corresponding to RSV-A (Table S1) complexed in a 4 to 1 ratio to streptavidin tetramers (Strep-G) as a convenient carrier for efficient presentation to the immune system. Mice received two intramuscular immunizations three weeks apart with a dose corresponding to 25 µg peptide adjuvanted with 2% Adjuplex or formulation buffer for the control animals. Three weeks after the final immunization, serum samples were obtained and tested for RSV-A G CCD binding and neutralizing antibody responses. All Strep-G immunized mice developed G CCD binding antibody responses as measured by ELISA (Figure 1C), but these were not able to neutralize RSV in a VNA on immortalized cells (Figure 1D). In contrast, the pooled serum samples of Strep-G immunized mice showed potent RSV neutralization activity in an hAEC-based VNA, with a comparable level of neutralization activity to pooled serum samples of mice immunized with stabilized soluble trimeric preF protein adjuvanted with 100 µg Adju-Phos (Figure 1E).

### 3.2. AaLS-RSV G CCD Vaccine Design

**Figure 2:**
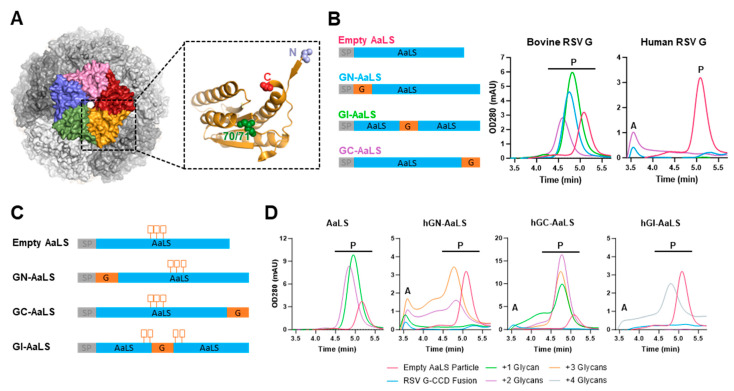
RSV G lumazine synthase nanoparticles. (**A**) Overall structure of self-assembling *Aquifex aeolicus* lumazine synthase particles. Monomeric subunits oligomerized into a pentamer are indicated by colors. Shades of gray indicate the organization of 12 pentamers forming 60-mer nanoparticles. The zoom-in depicts a single monomer with the locations indicated where peptides can be fused. (**B**) Analytical SEC traces of AaLS-G particles as determined in cell supernatant. Expression and oligomerization of N-terminal, C-terminal, or internal genetic fusion of RSV G CCD sequences to AaLS. The particle (‘P’) and aggregate (‘A’) peaks are indicated. The different line colors match the construct identifiers in the left panel. (**C**) Schematic of addition of glycan groups to AaLS-G nanoparticle designs. (**D**) Expression and oligomerization as assessed by analytical SEC of AaLS particles to which N-linked glycosylation sites were added. The particle (‘P’) and aggregate (‘A’) peaks are indicated.

To generate a G-based vaccine with biophysical properties that would be compatible with the manufacturing process, a self-assembling nanoparticle based on AaLS was used as a scaffold (Figure 2A). AaLS can be produced in mammalian expression systems, allows fusion of peptides to both termini as well as to an internal loop, and has high thermal and storage stability [31,38]. Since AaLS is a cytosolic protein in bacteria, we replaced the initiating methionine with a human CD5 signal peptide sequence to obtain a secreted particle. Peptide sequences corresponding to human or bovine RSV G CCD sequences, hereafter referred to as hG and bG, respectively, were fused to the AaLS N-terminus (GN-AaLS), C-terminus (GC-AaLS), or the internal loop of AaLS between positions 70 and 71 of the mature protein (GI-AaLS) (Figure 2B). DNA constructs encoding the G-AaLS variants were transiently expressed in Expi293F cells, and cell supernatants were harvested three days after transfection. Expression levels were assessed using analytical size exclusion chromatography (SEC) directly on the clarified supernatants and compared with an unmodified AaLS particle (Figure 2B). Analytical SEC was performed using an SRT column with a 500 Å pore size that enables separation of the correctly formed AaLS nanoparticles from aggregates and smaller structures. The empty AaLS particles could be detected as a distinct symmetrical peak eluting at 5.1 min retention time. AaLS fused to bG peptides showed strong particle expression, with bGN-AaLS and bGI-AaLS showing even higher expression than the empty particle, and an increased hydrodynamic radius as reflected by the shorter retention time (Figure 2B). In contrast, fusion of hG peptides to AaLS abolished particle formation completely, irrespective of the peptide placement, but instead led to aggregate formation as evidenced by the peak at 3.5 min retention time (Figure 2B). 

Although the overall structures of bovine and human RSV CCDs are identical [5,39], the isoelectric point (pI) of bG CCD (pI 5.5) is much lower than that of hG CCD (pI 9.2), which may explain the difference in expression and oligomerization of the fusion construct. Therefore, negatively charged linkers consisting of a repetitive ‘Gly-Glu’ sequence were introduced into the hG AaLS fusions to lower their pIs. While Western blot analysis of these charged linker designs in supernatant indeed showed increased expression (Figure S3A), the higher expression levels did not lead to measurable particle formation in analytical SEC (Figure S3B).

An alternative strategy to lower the pI and increase solubility of proteins is to introduce hydrophilic N-linked glycans. We designed hG-AaLS fusion constructs with additional N-linked glycosylation sites in the 70/71 loop of AaLS and evaluated expression levels and oligomerization status using analytical SEC. As naming convention, a number was added following the AaLS label denoting the number of N-linked glycosylation sites that were introduced, e.g., hGN-AaLS2 (Figure 2C,D). Glycan introduction boosted expression of empty particles and G-fused particles alike. Whereas hGN-AaLS required a minimum of two glycans for particle formation, a single glycan site sufficed for hGC-AaLS. However, designs with either two or three sites did give higher expression and less aggregate formation (Figure 2D). For the variant in which the RSV G CCD was inserted in the internal loop, four glycosylation sites, i.e., two motifs flanking the RSV G CCD sequence on each side, were required to yield high expression levels and particle formation. In conclusion, introduction of N-linked glycosylation sites proved an effective method to generate AaLS designs displaying the G CCD peptide of human RSV.

### 3.3. Purification and Biochemical Characterization of AaLS-RSV G Nanoparticles

Based on expression levels and oligomerization status in analytical SEC, particle variants hGN-AaLS2, hGC-AaLS2, and hGI-AaLS4 were selected for purification and detailed characterization. The nanoparticles were expressed at 300 mL scale in Expi293F cells and purified after 5 days using C-tag affinity chromatography followed by a SEC polishing step. 

**Figure 3:**
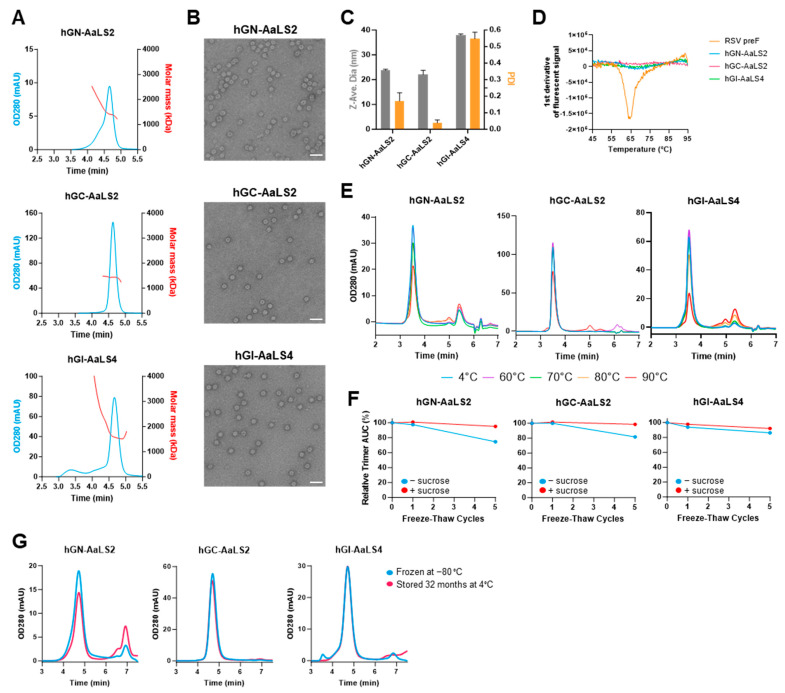
Characterization of purified lead AaLS-G nanoparticle candidates. (**A**) SEC-MALS traces of purified AaLS-G nanoparticles. OD280 UV traces appear in blue, and RI mass distribution is overlaid in red. AaLS-G nanoparticles eluted from the column at 4.6 min. (**B**) NS-TEM microscopy overview images showing the AaLS nanoparticles. Scale bar indicates 50 nm. (**C**) Particle size determination using dynamic light scatter (DLS). Z-average appears in grey, and the polydispersity index is noted in orange. Average of three measurements are plotted per sample; error bars indicate SD. (**D**–**F**) Particle stability in forced degradation experiments. (**D**) Melting temperature determined using DSF. The first derivative of the fluorescence signal is plotted. The lowest point on the graph corresponds to Tm50 values. (**E**) Analytical SEC OD280 UV traces of purified AaLS-G nanoparticles after 30 min heat destabilization at indicated temperatures compared to 4 °C control samples. AaLS-G nanoparticles eluted from the column at 3.5 min while other peaks correspond to degradation products. (**F**) Analytical SEC after indicated number of freeze–thaw cycles. Graph depicts area under the curve of the trimer peak after 1x and 5x freeze–thaw cycles as a trimer percentage relative to the 4 °C control sample. (**G**) Particle stability after storage of purified AaLS-G nanoparticles at 4 °C for 32 months as analyzed using analytical SEC. As a reference, freshly thawed material stored at −80 °C was used.

Analytical SEC of the purified hGC-AaLS2 particle showed a sharp peak with a consistent molar mass of 1450 kDa, while the hGN-AaLS2 and hGI-AaLS4 particle peaks were less uniform and showed the presence of higher molecular weight species (Figure 3A). Negative stain transmission electron microscopy (ns-TEM) revealed monodispersed, homogeneous spherical particles for all three variants of the expected size of approximately 22 nM (Figure 3B). The particle diameter was confirmed for hGN-AaLS2 and hGC-AalS2 using dynamic light scattering (DLS), yielding an average diameter of 23.8 and 22.0 nm respectively. Both designs exhibited a polydispersity index (PDI) of <0.3, indicative of a homogeneous particle population with GC-AaLS2 showing the lowest PDI, supporting the observed quality obtained by analytical SEC analysis (Figure 3C). The internal fusion design hGI-AaLS4 gave a larger diameter of 38.0 nm with DLS, which may be due to the presence of the higher molecular weight species that were also observed in analytical SEC (Figure 3C). 

Next, the purified particles were tested for thermal and storage stability. Differential scanning fluorimetry (DSF) did not show any denaturation event up to 95 °C, in contrast to a stabilized RSV preF protein [40] with a Tm50 of 64.6 °C (Figure 3D). Next, particles were incubated at increasing temperatures of 4, 60, 70, 80, and 90 °C for a period of 30 min, followed by analytical SEC (Figure 3E). All particles remained fully stable up to 60 °C. hGN-AalS2 exhibited a minor loss at 70 °C, while hGCI-AaLS4 and hGC-AaLS2 remained fully intact at 70 °C and 80 °C, respectively. Interestingly, even after exposure to a temperature of 90 °C, for all three designs, at least part of the particles remained intact. Next, we tested the stability of the particles after 1× or 5× freeze–thaw cycles in presence or absence of 5% *v*/*v* sucrose (Figure 3F). All three particles were highly resilient to a 1x freeze–thaw cycle, irrespective of the presence of sucrose. When exposed to 5× freeze–thaw cycles, in absence of sucrose, 75%, 82%, and 86% of particle remained for hGN-AaLS2, hGC-AaLS2, and hGI-AaLS4, respectively. These numbers increased to 95%, 99%, and 92%, respectively, when sucrose was added (Figure 3F). Finally, we determined long-term storage stability by incubating the purified particles at 4 °C for 32 months, followed by analytical SEC using freshly thawed material stored at −80 °C as reference (Figure 3G). Whereas hGN-AaLS2 showed some decrease in the particle peak concomitant with the appearance of smaller species, the hGC-AaLS2 and hGCI-AaLS4 particles fully retained their particle content. In conclusion, though all RSV G AaLS nanoparticles demonstrated excellent manufacturability characteristics and remarkable stability in forced degradation experiments, the hGC-AaLS2 design was determined to have the highest expression level and stability (Figure 2D and Figure 3).

### 3.4. AaLS-RSV G CCD Nanoparticles Elicit Potent Neutralizing Antibody Response In Vivo

**Figure 4:**
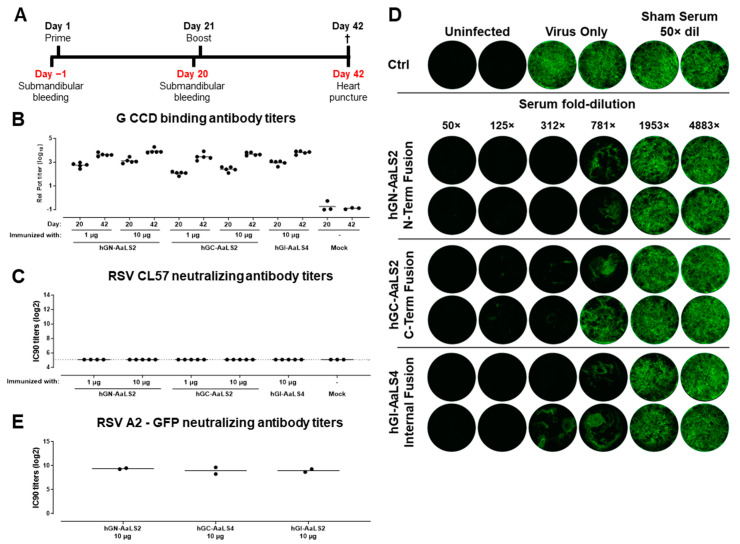
Preclinical evaluation of AaLS-G nanoparticle. (**A**) Experimental design of the mouse immunogenicity study. Mice were immunized with 1 or 10 µg AaLS-G nanoparticles displaying RSV G CCD peptide adjuvanted with 2% Adjuplex or formulation buffer at day 0 and 21 (*n* = 3 or 5, respectively). (**B**) RSV G CCD binding IgG antibody titers were determined by ELISA using immobilized CCD peptides and displayed as log10 relative potency (rel pot). (**C**) RSV A neutralizing antibody titers determined with VNAs using Firefly Luciferase (FFL) labeled RSV A strain CL57 and A549 cells. Titers are displayed as Log2 of the 90% FFL inhibitory serum dilution (IC90) in serum samples isolated at day 20 and/or day 42. Bars represent mean responses per group; the dashed line indicates the lower limit of quantification (LLOQ) of the assay. (**D**,**E**) RSV A2-GFP neutralizing antibody titers of serum from 10 µg dosed mice, isolated on day 42 and pooled per immunogen, in hAEC-based VNAs. (**D**) Depicted are GFP signals of each insert, 96 h post-infection. Infected cells turn green by reporter protein expression. (**E**) hAEC VNA titers shown as log2 values of the 90% inhibitory concentration (IC90). Bars indicate mean values of two technical replicates depicted by dots.

To assess the ability of AaLS-RSV G CCD NPs to induce RSV G CCD binding and RSV neutralizing antibody responses, mice were immunized intramuscularly twice with a 3-week interval with 1 or 10 µg AaLS-G NP adjuvanted with 2% Adjuplex. On day 20, blood was collected. On day 42, the mice were sacrificed, and final bleeds were collected (Figure 4A).

RSV G CCD binding and RSV neutralizing antibody titers were measured. Robust G CCD binding antibody titers were induced after a single immunization and were further increased after the second immunization (Figure 4B). hGN-AaLS2 induced overall the highest G CCD binding antibody titers, which were significantly higher compared to the other two particles after the first immunization (*p* < 0.0001, Tobit model) and significantly higher than that induced by hGC-AaLS2 particles after the second immunization (*p* = 0.0322, Tobit model).

In concordance with earlier data obtained with RSV G mAbs, the polyclonal sera against the AaLS-RSV G NPs were unable to neutralize RSV in a VNA using A549 cells (Figure 4C). Serum samples were therefore tested in a VNA using hAECs (Figure 4D,E). In hAEC VNAs, sera from mice immunized twice with the three different adjuvanted AaLS-RSV G CCD nanoparticles showed strong neutralization activity with no clear difference between the particles. Full viral breakthrough occurred only with almost 2000-fold diluted sera, underscoring the potency of the induced RSV G CCD directed neutralizing antibody titers. In conclusion, all three tested particles were effective in inducing neutralizing antibody responses in mice, as assessed in hAEC VNAs, irrespective of the placing of the peptide.

## 4. Discussion

In recent years, tremendous progress has been made in the development of RSV vaccines, with two vaccines now commercially available. These vaccines use the F protein stabilized in the prefusion conformation as a vaccine immunogen and are either based on RSV-A or a combination of RSV-A and RSV-B [13,14]. Although both vaccines have shown high efficacy against contemporary RSV-A and RSV-B strains, the F protein sequence is subject to drift mutations, possibly hampering preF-based vaccine efficacy in the future [41]. A solution would be to monitor the accumulation of mutations in the F protein and to update the preF-based RSV vaccines to match the circulating strains. This labor-intensive approach is currently used for influenza vaccines and has encouraged the development of universal influenza vaccines that protect against current and future seasonal strains as well as potential pandemic strains [42,43,44,45]. Such a universal approach would also be appealing in the RSV vaccine field. It is believed that the CCD of the RSV G protein is involved in receptor binding and is therefore conserved within the RSV A and RSV B subtypes, making this region an attractive vaccine target. 

Several vaccine approaches based on RSV G have been investigated, utilizing the CCD as a stand-alone antigen or as an add-on component, co-formulated with an RSV F vaccine [21,46,47,48,49,50,51,52,53,54,55,56]. Multiple strategies have been explored to overcome the limited immunogenicity of the CCD peptide, including multimerization of short peptides and display on synthetic, as well as self-assembling nanoparticles. Anti-G CCD antibody titers are induced in these studies as demonstrated by ELISA, and in vivo challenge models in mice or cotton rats have demonstrated the efficacy of G-based vaccine candidates by measuring reduced lung viral loads or reduced lung pathology. It is believed that the induced G antibodies exert their effect through effector functions, such as complement activation. Determining the direct neutralizing potency of these G binding antibodies in vitro is possible, but contingent on using cell systems that reiterate human airway tissue, e.g., hAEC cultures. While RSV F targeted virus neutralization can be measured in immortalized cells, such as A549 or HeLa, the neutralizing capacity of RSV G-binding antibodies cannot be measured in such assays. Advancements in upscaling of 3D culturing methods have made it possible to employ fully differentiated human epithelial airway cells (hAECs) to assess in vivo virus neutralization of respiratory viruses. hAEC cultures of nasal origin closely mimic the natural site of infection and, as such, carry the in vivo cell surface receptor for RSV [18,57]. 

Here, we confirmed the direct neutralizing potency of two anti-RSV G-CCD binding monoclonal antibodies using hAEC cultures and compared them with anti-F antibodies. In parallel, full viral breakthrough at all tested antibody concentrations was demonstrated in A549 and HeLa cells. These results indicate a distinct difference in target cell binding between hAEC and HeLa cells for RSV. Indeed, we and others have shown that RSV attachment and entry in immortalized cells are mediated by binding to cell surface heparan sulfate structures (Figure S2). Here, we showed that RSV is not capable of infecting HeLa cells in which ß1,4-galactosyltrasferase 7 (B4GALT7) has been knocked out. B4GALT7 is one of the key glycan modifying enzymes, catalyzing linkage between proteins and glycosaminoglycans in proteoglycans, such as heparan sulfate. B4GALT7 knock out prevents the formation of heparan sulfate on the cell surface of HeLa cells, rendering them unsusceptible to RSV attachment and subsequent infection. Heparan sulfate is ubiquitously present on immortalized cell lines and may explain the wide range of cells lines that RSV can infect in vitro. However, heparan sulfate is reportedly absent from human respiratory tissue, and heparan sulfate-dependent entry likely does not represent the in vivo entry route. 

Establishing hAEC VNA as the representative assay with more predictive value than immortalized cells, we compared for the first time the neutralizing potential of antibodies against G and F side-by-side. Interestingly, the two randomly selected mAbs against G outperformed mAbs against F in hAEC-based neutralization. Next, the potential of a G-based vaccine was demonstrated by immunizing mice with human RSV G CCD streptavidin conjugates that induced high ELISA titers and neutralized the virus in hAEC VNA (Figure 1C,E). 

The conversion of peptides into an immunogenic vaccine platform presents inherent challenges, which we addressed by fusion of G peptides to the AaLS scaffold, derived from the extremophile *Aquifex aeolicus*. Here, we achieved a symmetrical and repetitive display conducive to enhanced cross-linking of B cell receptors, B cell activation, and proliferation. This multivalent antigen presentation scaffold of AaLS, enhanced by the introduction of N-linked glycosylation sites, exhibited remarkable thermal and storage stability. Importantly, the high expression levels of the nanoparticles are likely compatible with industrial-scale purification processes using SEC. Preclinical evaluation in a naïve mouse model demonstrated the nanoparticles’ capacity to elicit RSV neutralizing antibodies, as further confirmed in neutralization assays on primary, fully differentiated human airway epithelial cell cultures. Although we used Adjuplex as an adjuvant in our preclinical experiments in naïve mice, it remains to be seen if adjuvants are required in a clinical setting where subjects are most likely RSV exposed. 

In conclusion, our findings underscore the significance of RSV G in the viral lifecycle and present a compelling next-generation RSV vaccine candidate characterized by favorable manufacturability and immunogenic properties. This candidate holds promise for independent use or in synergy with existing F-based vaccines, marking a significant advancement in the pursuit of effective RSV immunization strategies.

## 5. Patents

MJGB and JPML are co-inventors on a patent describing the AaLS-G nanoparticles.

## Data Availability

All data to understand and assess the conclusions of this research are available in the main text and Supplemental Materials. The raw data that support the findings of this study are available from the corresponding authors upon reasonable request.

## Supplementary Materials

The following supporting information can be downloaded at: https://www.mdpi.com/article/10.3390/vaccines12030294/s1, Table S1: Peptides used for streptavidin coupling and ELISA. Figure S1: Neutralizing potency of anti-RSV F and G monoclonal antibodies on HeLa cells using RSV A2-GFP reporter virus. Figure S2: Wildtype and B4GALT7 KO HeLa cells infected with RSV A2-GFP. Figure S3: Optimization of AaLS-G nanoparticle expression.
