# Supplementary material for: Design and Preclinical Evaluation of a Nanoparticle Vaccine against Respiratory Syncytial Virus Based on the Attachment Protein G
Source: Vaccines (Basel). 2024 Mar 12;12(3):294. doi: 10.3390/vaccines12030294 (PMC10975092; doi:10.3390/vaccines12030294)
Supplement: Supplementary file 1 [file vaccines-12-00294-s001.zip › vaccines-2897920-supplementary.pdf]

Table S1

ID	*N*-terminus	RSV G-CCD amino acid sequence	*C*-Terminus
hRSV1	Biotin	KQRQNKPPNKPNNDFHFEVFNFVP**C**SI**C**SNNPT**C**WAI**C**KRIPNKKPGKKTTTKPTKK	NH_2
hRSV2	Biotin	NKPNNDFHFEVFNFVP**C**SI**C**SNNPT**C**WAI**C**KRIPNKKPGKK	NH_2
bRSV	Biotin	NPSESNPPENHQDHNNSQTLPHVP**C**ST**C**EGNPA**C**LSL**C**QIGPESASSRAPTITLKKI	NH_2

Table S1. Peptides used for streptavidin coupling and ELISA.

Fig. S1

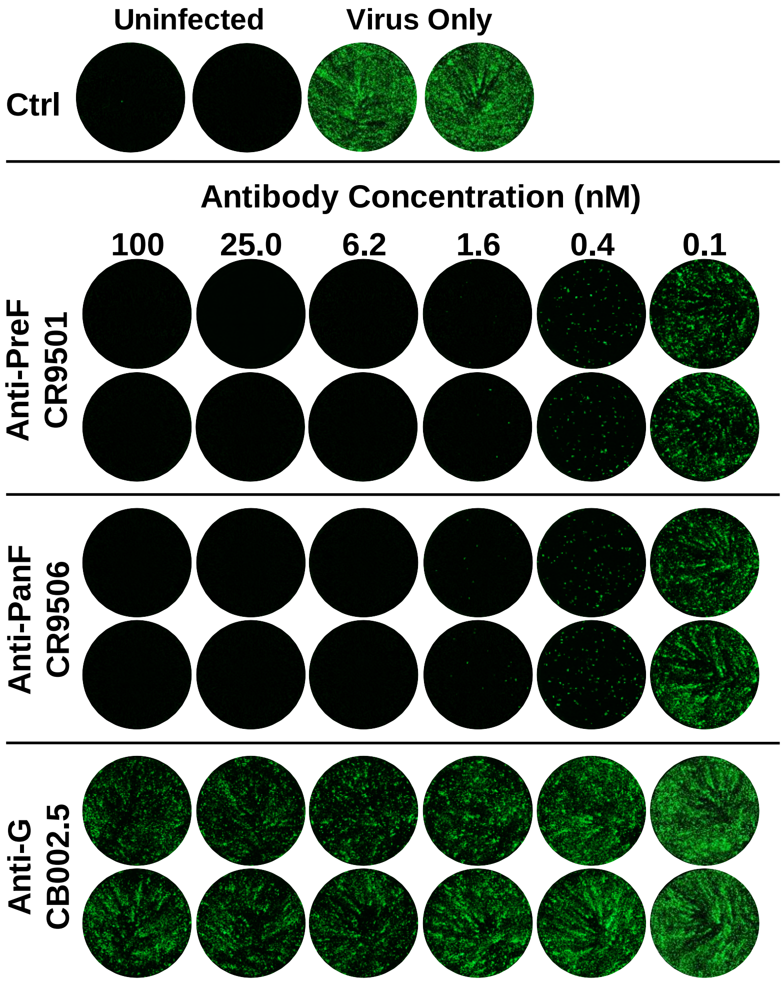

Figure S1. Neutralizing potency of anti-RSV F and G monoclonal antibodies on RSV A2-GFP reporter gene virus determined on HeLa cells.

Fig. S2

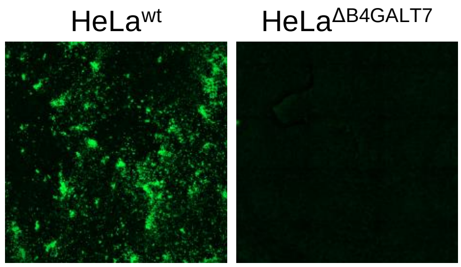

Figure S2. Wildtype and B4GALT7 KO HeLa cells were infected with RSV-GFP and imaged after 3 days.

Fig. S3

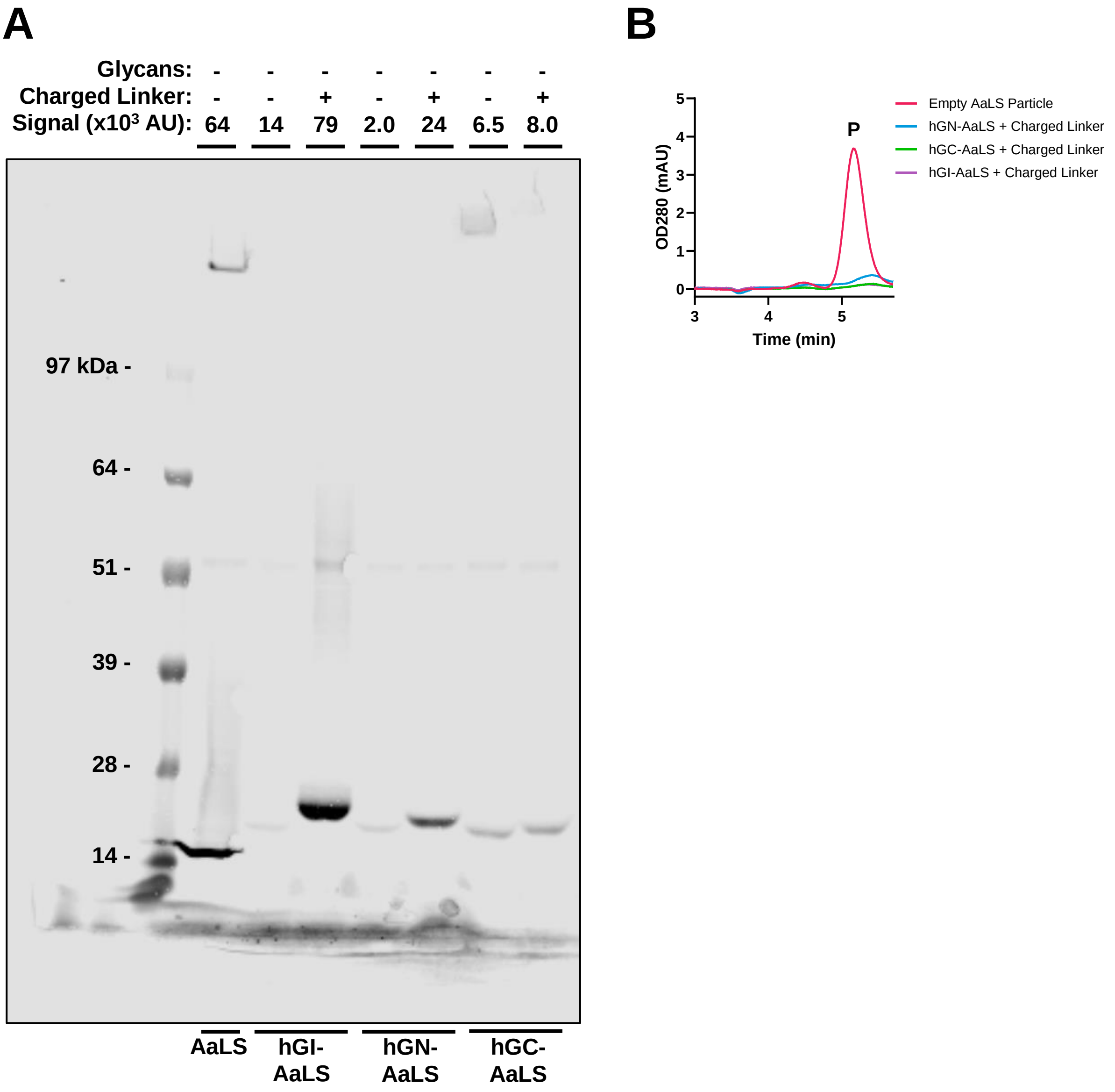

Figure S3. Optimization of AaLS-G nanoparticle expression. **(A)** Western blot analysis of AaLS-G particle designs incorporating charged linkers in supernatant of Expi293F cells. The addition of glycan motifs and charged linkers as well as the fluorescence intensity of the band between 14 and 28 kDa in AU are indicated for each tested design. **(B)** Expression and oligomerization of N-terminal, C-terminal or internal genetic fusion of RSV G CCD designs of (A). Analytical SEC traces illustrate the effects of introducing charged linked on expression levels in supernatant.
